# Bcl-xL inhibition by molecular-targeting drugs sensitizes human pancreatic cancer cells to TRAIL

**DOI:** 10.18632/oncotarget.5881

**Published:** 2015-10-19

**Authors:** Yoko Hari, Nanae Harashima, Yoshitsugu Tajima, Mamoru Harada

**Affiliations:** ^1^ Department of Surgery, Shimane University Faculty of Medicine, Shimane, Japan; ^2^ Department of Immunology, Shimane University Faculty of Medicine, Shimane, Japan

**Keywords:** pancreatic cancer, TRAIL, apoptosis, Bcl-2, Bcl-xL

## Abstract

Tumor necrosis factor (TNF)-related apoptosis-inducing ligand (TRAIL) induces apoptosis in various types of cancer cells without damaging normal cells. However, in terms of pancreatic cancer, not all cancer cells are sensitive to TRAIL. In this study, we examined a panel of human pancreatic cancer cell lines for TRAIL sensitivity and investigated the effects of Bcl-2 family inhibitors on their response to TRAIL. Both ABT-263 and ABT-737 inhibited the function of Bcl-2, Bcl-xL, and Bcl-w. Of the nine pancreatic cancer cell lines tested, six showed no or low sensitivity to TRAIL, which correlated with protein expression of Bcl-xL. ABT-263 significantly sensitized four cell lines (AsPC-1, Panc-1, CFPAC-1, and Panc10.05) to TRAIL, with reduced cell viability and increased apoptosis. Knockdown of Bcl-xL, but not Bcl-2, by siRNA transfection increased the sensitivity of AsPC-1 and Panc-1 cells to TRAIL. ABT-263 treatment had no effect on protein expression of Bcl-2, Bcl-xL, or c-FLIPs. In Panc-1 cells, ABT-263 increased the surface expression of death receptor (DR) 5; the NF-κB pathway, but not endoplasmic reticulum stress, participated in the increase. In xenograft mouse models, the combination of TRAIL and ATB-737 suppressed the *in vivo* tumor growth of AsPC-1 and Panc-1 cells. These results indicate that Bcl-xL is responsible for TRAIL resistance in human pancreatic cancer cells, and that Bcl-2 family inhibitors could represent promising reagents to sensitize human pancreatic cancers in DR-targeting therapy.

## INTRODUCTION

Apoptosis is primarily induced in cancer cells through two major pathways: extrinsic and intrinsic [1, 2]. Fas ligand (FasL) and tumor necrosis factor (TNF)-related apoptosis-inducing ligand (TRAIL) can provide a death signal via the extrinsic apoptotic pathway. It is therapeutically important that only TRAIL can induce cancer cell death while causing almost no cytotoxicity to normal cells [3]. TRAIL receptors consist of positive and negative receptors; death receptor (DR)4 and DR5 provide pro-apoptotic signaling, whereas decoy receptor (DcR)1 and DcR2 competitively inhibit apoptotic signaling [3]. Normal cells show TRAIL resistance with their preferential expression of DcRs [4]. Based on these lines of evidence, TRAIL and its DRs are expected to be promising target molecules in anti-cancer therapy [5, 6].

Many molecules are involved in apoptosis [7]. Among them, the Bcl-2 family of molecules is involved in intrinsic apoptosis via mitochondria [8, 9]. The family of Bcl-2-related anti-apoptotic proteins includes Bcl-2, Bcl-xL, Bcl-w, and Mcl-1. These proteins inhibit cell death by sequestering the pro-apoptotic proteins Bax and Bak and by preventing their oligomerization [10–13]. An increase in Bcl-2 expression protects cancer cells from apoptosis [14, 15], and the elevated expression of Bcl-2 and Bcl-xL has been frequently observed in a variety of cancers [9]. Thus, the inhibition of Bcl-2 and/or Bcl-xL is hypothesized to potentiate the effect of chemotherapy and, consequently, several Bcl-2 family inhibitors have been developed. ABT-737 is a small molecule inhibitor of Bcl-2, Bcl-xL, and Bcl-w [16]. ABT-263 (Navitoclax) is a clinically approved orally bioavailable inhibitor with the same specificity as ABT-737 [17, 18]. ABT-199 is a new, orally bioavailable inhibitor that inhibits Bcl-2 and Bcl-w, but not Bcl-xL [19]. Several reports have demonstrated the efficacy of these inhibitors against hematological malignancies as well as solid tumors [20–26].

In this study, we investigated the effects of the Bcl-2 family inhibitors on TRAIL sensitivity using a panel of human pancreatic cancer cell lines. Of nine pancreatic cancer cell lines, six showed either no or low sensitivity to TRAIL, and this resistance was positively correlated with the protein expression of Bcl-xL. ABT-263 significantly sensitized four cell lines to TRAIL, with reduced cell viability and increased apoptosis. Additional analysis of AsPC-1 and Panc-1 cell lines revealed that the inhibition of Bcl-xL, but not Bcl-2, increased the TRAIL sensitivity of these cells. In xenograft mouse models, the combination of TRAIL and ATB-737 exerted a significant antitumor effect on AsPC-1 and Panc-1. These results indicate that Bcl-xL is responsible for TRAIL resistance in human pancreatic cancer cells, and that the Bcl-2 family of inhibitors, such as ABT-263 and ABT-737, could be promising reagents to sensitize human pancreatic cancer cells to DR-targeting therapy.

## RESULTS

### Varied TRAIL sensitivity of human pancreatic cancer cell lines

**Figure 1:**
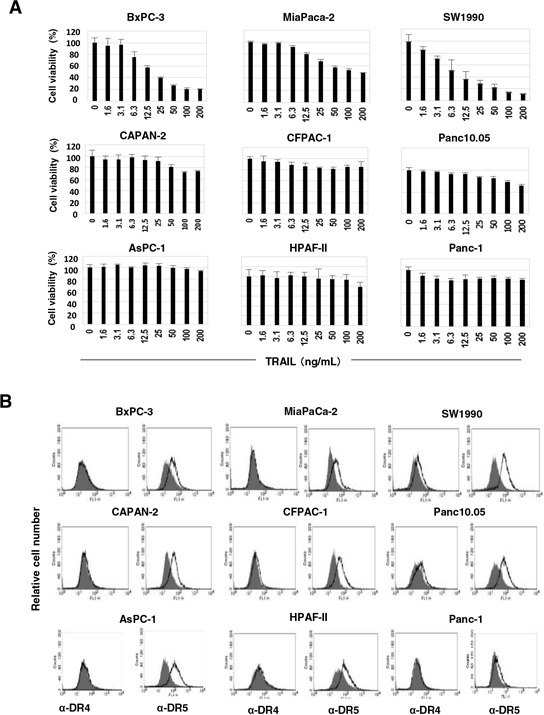
TRAIL sensitivity and expression of DRs on nine pancreatic cancer cell lines **A.** Nine human pancreatic cancer lines were cultured in the presence of TRAIL (ng/mL). After 48 h, cell viability (%) was determined by the WST-8 assay. The data shown represent the mean of three wells. **B.** The expression of DR4 and DR5 on nine cell lines was examined by flow cytometry. The line represents staining with mAbs specific to either DR4 or DR5, followed by a FITC-conjugated secondary antibody. The solid gray represents staining with FITC-conjugated anti-mouse IgG alone.

Initially, we examined the sensitivity of nine human pancreatic cancer cell lines to TRAIL. The viability of three cell lines (BxPC-3, MiaPaCa-2, and SW1990) decreased in the presence of TRAIL in a dose-dependent manner, whereas the other six lines showed no or low sensitivity to TRAIL (Figure 1A). We also examined the expression of the TRAIL receptors on these cells. Although both SW1990 and Panc10.05 were slightly positive for DR4, the other seven cell lines were negative for this receptor. All nine cell lines were positive for DR5 (Figure 1B). DcR1 and DcR2 were not detected on all cell lines (Supplementary Figure S1). These results indicated that DR5 expression does not reflect the TRAIL sensitivity of the human pancreatic cancer cell lines and that other regulatory mechanisms determine their TRAIL sensitivity.

### Combination effect of TRAIL and ABT-263 on TRAIL-insensitive human pancreatic cancer cell lines

**Figure 2:**
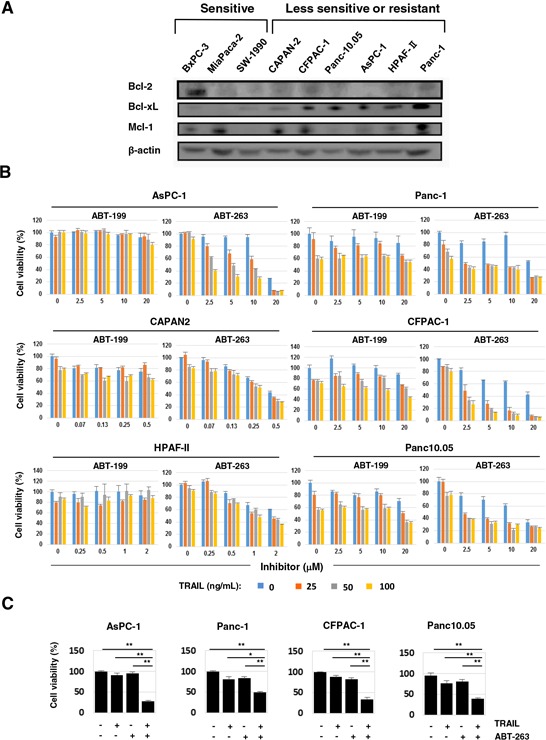
ABT-263 sensitized four pancreatic cancer cell lines to TRAIL **A.** The nine pancreatic cancer cell lines were examined for their expression of Bcl-2, Bcl-xL, and Mcl-1 by immunoblot. β-Actin was used as a loading control. **B.** Six pancreatic cancer cell lines were cultured with the indicated concentrations of TRAIL (ng/mL) with either ABT-199 or ABT-263 (μM). After 48 h, cell viability (%) was determined using the WST-8 assay. The results are shown as the mean ± SD of three wells. **C.** Selected results of four cell lines that were cultured with TRAIL and/or ABT-263 are shown. The following doses were used: TRAIL (100 ng/ml) and ABT-263 (10 μM) for AsPC-1 cells, and TRAIL (50 ng/ml) and ABT-263 (2.5 μM) for Panc-1, CFPAC-1, and Panc10.05 cells. *P < 0.05, **P < 0.01.

Before testing the combination effect of TRAIL and the Bcl-2 family inhibitors, we examined the expression of anti-apoptotic Bcl-2 family proteins (Bcl-2, Bcl-xL, and Mcl-1) in nine cancer cell lines (Figure 2A). The expression levels of Bcl-2 and Mcl-1 varied among the nine cancer cell lines, suggesting that it was not contributing to TRAIL resistance. In contrast, a higher expression of Bcl-xL was detected in the six TRAIL-insensitive cell lines compared with the three TRAIL-sensitive cell lines (BxPC-3, MiaPaca-2, and SW-1990). We examined the antitumor effect of TRAIL on the six TRAIL-insensitive pancreatic cancer cell lines when combined with orally bioavailable Bcl-2 inhibitors, ABT-199 or ABT-263 (Figure 2B). ABT-199 is an inhibitor of Bcl-2 and Bcl-w, and ABT-263 is an inhibitor of Bcl-2, Bcl-xL, and Bcl-w [17–19]. In contrast to the no-combination effect of TRAIL and ABT-199, ABT-263 significantly augmented the TRAIL sensitivity of four of the cancer cell lines (AsPC-1, Panc-1, CFPAC-1, and Panc10.05). Representative results are shown in Figure 2C. The two cell lines, CAPAN2 and HPAF-II, were highly sensitive to ABT-263 alone, and a dose of 0.5 or 2 μM reduced the cell viability of these lines by approximately 50%. The relationship between Bcl-xL expression and TRAIL sensitivity and the specificity of ABT-263 and ABT-199 suggest that Bcl-xL is responsible for TRAIL resistance in human pancreatic cancer cells and that inhibition of Bcl-xL by ABT-263 is a useful way to sensitize human pancreatic cancer cells to TRAIL.

### Caspase-dependent apoptosis in human pancreatic cancer cells using a combination of TRAIL and ABT-263

**Figure 3:**
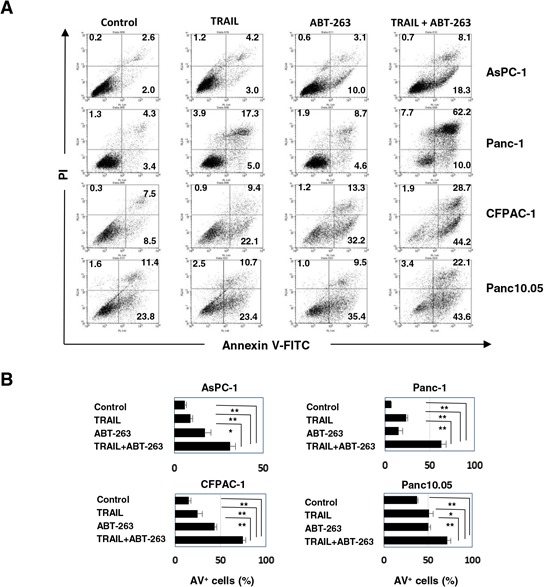
Apoptosis in pancreatic cancer cell lines treated with the combination of TRAIL and ABT-263 **A.** Four pancreatic cancer cell lines were cultured with TRAIL and/or ABT-263 for 48 h. After staining with Annexin V-FITC/PI, flow cytometric analysis was performed. The numbers represent the proportions of each subset. **B.** The percentages of Annexin V (AV)^+^ cells were calculated. All data points shown represent the mean of three culture wells. The following doses were used: TRAIL (25 ng/ml) and ABT-263 (2.5 μM) for AsPC-1 cells, TRAIL (100 ng/ml) and ABT-263 (5 μM) for Panc-1 cells, and TRAIL (50 ng/ml) and ABT-263 (5 μM) for CFPAC-1 and Panc10.05 cells. *P < 0.05, **P < 0.01.

**Figure 4:**
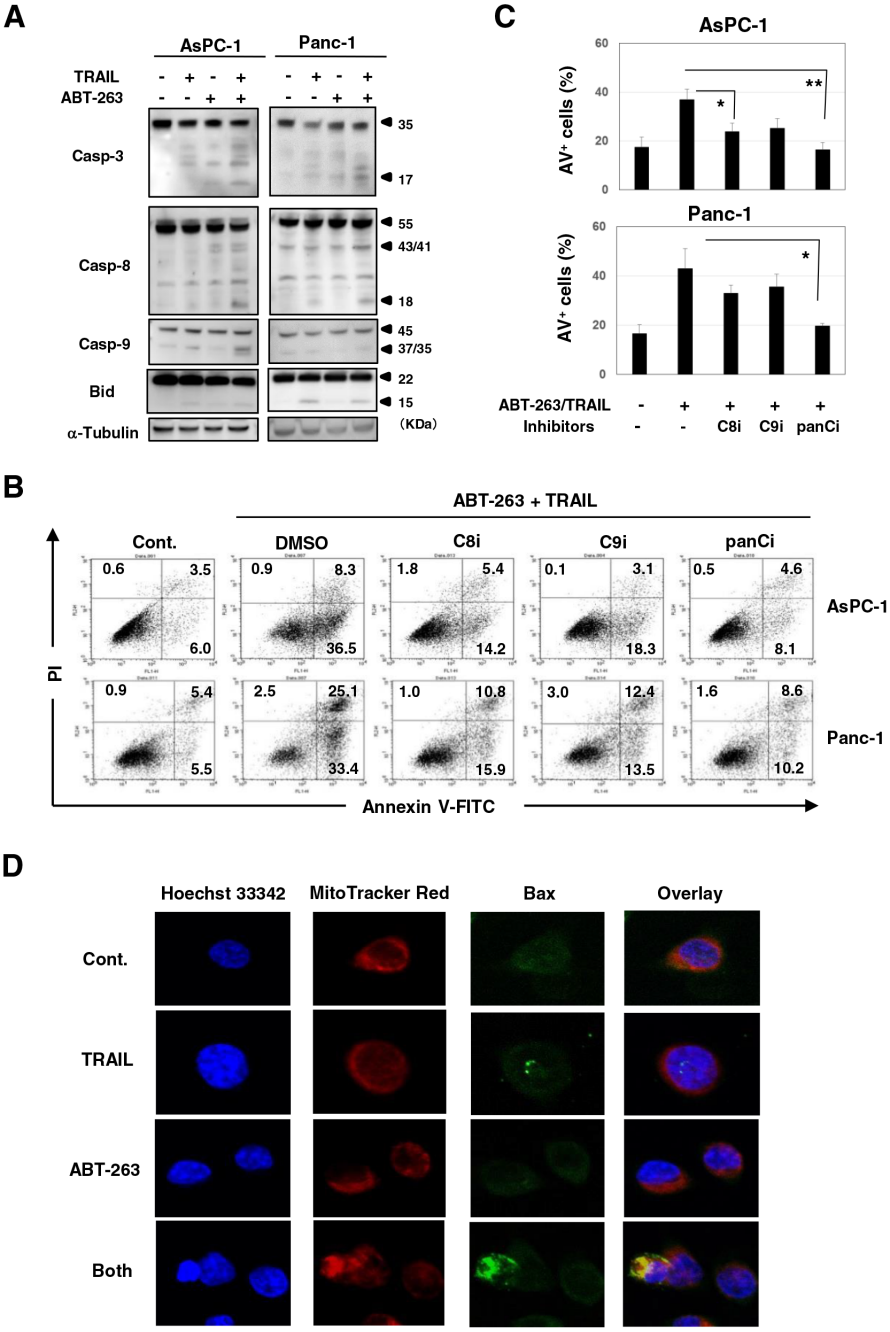
Caspase-dependent apoptosis of AsPC-1 and Panc-1 cells after combination treatment with TRAIL and ABT-263 **A.** Cancer cells were treated with TRAIL and/or ABT-263. After 24 h, the cells were harvested and cell lysates were assayed for their expression of caspase-3, −8, −9, and Bid by immunoblot. α-Tublin was used as a loading control. The following doses were used: TRAIL (25 ng/ml) and ABT-263 (1 μM) for AsPC-1 cells, TRAIL (50 ng/ml) and ABT-263 (5 μM) for Panc-1 cells. **B.** Cancer cells were treated with TRAIL (25 ng/mL) and ABT-263 (1 μM) in the presence of several caspase inhibitors for 48 h. After staining with Annexin V-FITC/PI, flow cytometric analysis was performed. The numbers represent the proportions of each subset. panCi, pan-caspase inhibitor; C9i, caspase-9 inhibitor; C8i, caspase-8 inhibitor. As the vehicle control, the same volume of DMSO was added. **C.** The percentages of Annexin V (AV)^+^ cells were calculated. All data points shown represent the mean of three culture wells. *P < 0.05, **P < 0.01. **D.** AsPC-1 cells were cultured with TRAIL (25 ng/mL) and/or ABT-263 (1 μM) for 12 h. After incubation with Hoechst 33342 and MitoTracker Red for 30 min, cells were stained with anti-Bax antibody followed by Alexa Fluor 488-conjugated anti-rabbit IgG F(ab’)2 fragment. Confocal imaging revealed nuclei (blue), mitochondria (red), and Bax (green). Yellow represents Bax that localized to the mitochondria.

We determined whether the effect seen with a combination of TRAIL and ABT-263 was the result of enhanced apoptosis in cancer cells. Compared with either TRAIL or ABT-263 alone, the combination increased the percentage of Annexin V^+^ cells in four of the pancreatic cancer cell lines (Figure 3A and 3B). Additional analysis was performed by focusing on two cell lines, AsPC-1 and Panc-1. The combination of TRAIL and ABT-263 increased the expression of cleaved caspase-3, caspase-8, and caspase-9 in AsPC-1 cells (Figure 4A). In terms of Panc-1 cells, the combination increased the expression of cleaved caspase-3 and caspase-8, but no clear cleavage of caspase-9 was observed. Bid is the link between extrinsic and intrinsic apoptosis [3]. TRAIL treatment slightly induced the expression of truncated Bid in both cell lines, but the addition of ABT-263 failed to enhance the TRAIL-induced expression of truncated Bid. Apoptosis by combination treatment of TRAIL and ABT-263 was inhibited by the addition of caspase-8, caspase-9, or pan-caspase inhibitors (Figure 4B and 4C). Given that Bax oligomerization and translocation is essential for intrinsic apoptosis [10, 12] and that some small molecules sensitize pancreatic cancer cells to TRAIL via Bax oligomerization and translocation [27], we examined the expression and localization of Bax in treated cancer cells. As a result, Bax localized to the mitochondria only when cancer cells were treated with both TRAIL and ABT-263 (Figure 4D) (Supplementary Figure S2). These results indicate that the combination of TRAIL and ABT-263 can induce caspase-dependent apoptosis in TRAIL-insensitive pancreatic cancer cell lines with Bax translocation to the mitochondria.

### Bcl-xL inhibition can sensitize TRAIL-resistant pancreatic cancer cells to TRAIL

**Figure 5:**
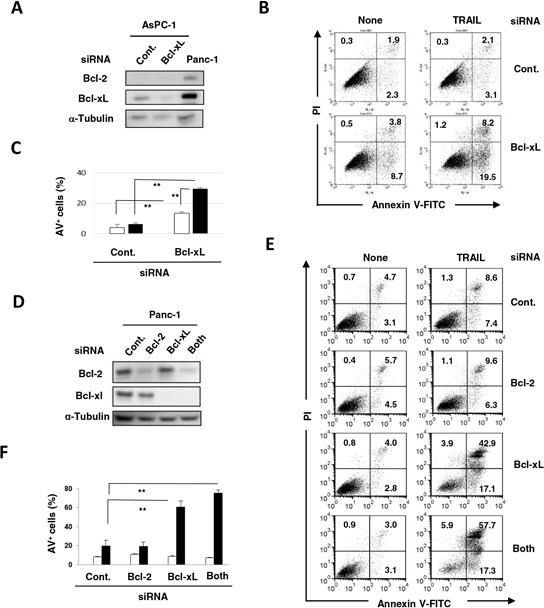
Protective role of Bcl-xL in TRAIL-treated AsPC-1 and Panc-1 cells **A.** AsPC-1 cells were transfected with the indicated siRNA and analyzed for expression of Bcl-2 and Bcl-xL by immunoblot. α-Tubulin was used as a loading control. **B.** siRNA-transfected AsPC-1 cells were cultured with TRAIL (25 ng/mL). After 48 h, cells were stained with FITC-conjugated Annexin V/PI and examined by flow cytometry. The numbers represent the proportions of each subset. **C.** The percentage of Annexin V (AV)^+^ cells was calculated. All data points shown represent the mean of three culture wells. The open and closed bars represent the results without and with TRAIL (25 ng/mL), respectively. **P < 0.01. **D.** Panc-1 cells transfected with the indicated siRNA were analyzed for their expression of Bcl-2 and Bcl-xL by immunoblotting. α-Tubulin was used as a loading control. **E.** siRNA-transfected Panc-1 cells were cultured with TRAIL (25 ng/mL). After 48 h, cells were stained with FITC-conjugated Annexin V/PI and examined by flow cytometry. The numbers represent the proportions of each subset. **F.** The percentage of Annexin V (AV)^+^ cells was calculated. All data points shown represent the mean of three culture wells. Open and closed bars represent the results without and with TRAIL (25 ng/mL), respectively. **P < 0.01.

The mechanism by which the combination of TRAIL with ABT-263 induced TRAIL sensitivity in TRAIL-resistant pancreatic cancer cells was investigated. Although AsPC-1 cells were negative for Bcl-2, transfection of Bcl-xL siRNA selectively reduced the Bcl-xL expression in AsPC-1 cells (Figure 5A). TRAIL significantly increased the percentage of Annexin V^+^ cells in Bcl-xL siRNA-transfected AsPC-1 cells compared with that of the control siRNA-transfected cells (Figure 5B and 5C). Panc-1 cells were positive for both Bcl-2 and Bcl-xL, and transfection of Bcl-2 or Bcl-xL siRNA selectively knocked down the respective molecule in Panc-1 cells (Figure 5D). Additionally, TRAIL significantly increased the percentage of Annexin V^+^ cells in Panc-1 cells that were pre-transfected with either Bcl-xL siRNA or both Bcl-xL and Bcl-2 siRNA (Figure 5E and 5F). These results indicated that Bcl-xL is responsible for the TRAIL resistance of AsPC-1 and Panc-1 cells and that inhibition of this anti-apoptotic protein can effectively sensitize TRAIL-resistant pancreatic cancer cells to TRAIL.

### Involvement of the NF-κB pathway, but not ER stress, in the increased expression of DR5 on ABT-263-treated Panc-1 cells

**Figure 6:**
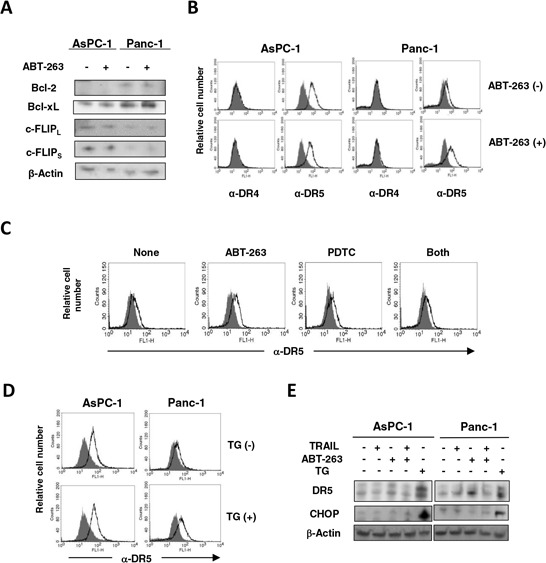
Increased expression of DR5 on ABT-263-treated Panc-1 cells **A.** AsPC-1 and Panc-1 cells were cultured with ABT-263 (2.5 μM), and the expression of Bcl-2, Bcl-xL, and c-FLIPs was examined by immunoblotting. β-Actin was used as a loading control. **B.** AsPC-1 and Panc-1 cells were cultured with ABT-263 (2.5 μM). After 24 h, expression of DR4 and DR5 was examined by flow cytometry. The line represents staining with mAb specific to either DR4 or DR5, followed by a FITC-conjugated secondary antibody. Solid gray represents staining with FITC-conjugated anti-mouse IgG alone. **C.** Similarly, Panc-1 cells were cultured with ABT-263 (2.5 μM) and/or PDTC (5 μM). After 24 h, expression of DR5 was examined by flow cytometry. **D.** AsPC-1 and Panc-1 cells were cultured with thapsigargin (TG) (5 μM). After 24 h, the expression of DR5 was examined by flow cytometry. **E.** AsPC-1 and panc-1 cells were cultured with TRAIL (25 ng/mL), ABT-263 (1 μM), or TG (5 μM). After 24 h, the expression of DR5 and CHOP was examined by immunoblotting. β-Actin was used as a loading control.

The mechanism by which ABT-263 sensitized pancreatic cancer cells to TRAIL was investigated. Treatment with ABT-263 showed no effect on the protein expression of Bcl-2 and Bcl-xL in AsPC-1 and Panc-1 cells (Figure 6A). c-FLIPs are known to be inhibitors of caspase-8 [28], whereas their expression was not changed by ABT-263 treatment. We examined the expression of DRs on these cells and observed that ABT-263 increased the DR5 expression on Panc-1 cells only (Figure 6B). The ABT-263 showed no effect on DR5 expression on CFPAC-1 and Panc10.05 cells, whose sensitivity to TRAIL was induced by combining TRAIL with ABT-263 (Figure 2B and 2C) (Supplementary Figure S3). Alternatively, ABT-737 has been reported to increase the DR5 expression on prostate, renal, and lung cancer cell lines at a transcriptional level as a result of stimulation of NF-κB activity [29]. Therefore, we examined the effect of pyrrolidine dithiocarbamate (PDTC), an NF-κB inhibitor [30], and found that PDTC inhibited an increase in the DR5 expression on ABT-263-treated Panc-1 cells (Figure 6C). Additionally, because recent reports have revealed that endoplasmic reticulum (ER) stress can increase the DR5 expression on/in human cancer cells [31, 32], we examined the effect of thapsigargin, an ER stress inducer [33], on the expression of DR5. As shown in Figure 6D, treatment with thapsigargin increased DR5 expression on Panc-1 cells but not on AsPC-1 cells. Additionally, immunoblot experiments revealed that thapsigargin increased the expression of DR5 as well as CCAAT/enhancer-binding protein homologue protein (CHOP), an indicator of ER stress [34], on AsPC-1 and Panc-1 cells (Figure 6E). In contrast, ABT-263 increased the expression of DR5 only in Panc-1 cells in the absence of the increased expression of CHOP. ABT-263 treatment increased the expression of DR5 in Panc-1 cells, but the combination of ABT-263 and TRAIL unexpectedly reduced the expression of DR5. We hypothesized that this result was due to the preferential apoptosis of TRAIL-treated Panc-1 cells that express DR5 at higher levels. These results indicate that the NF-κB pathway, but not ER stress, was involved in an increase in DR5 expression on ABT-263-treated Panc-1 cells.

### The combination effect of TRAIL and ABT-737 in xenograft mouse models

**Figure 7:**
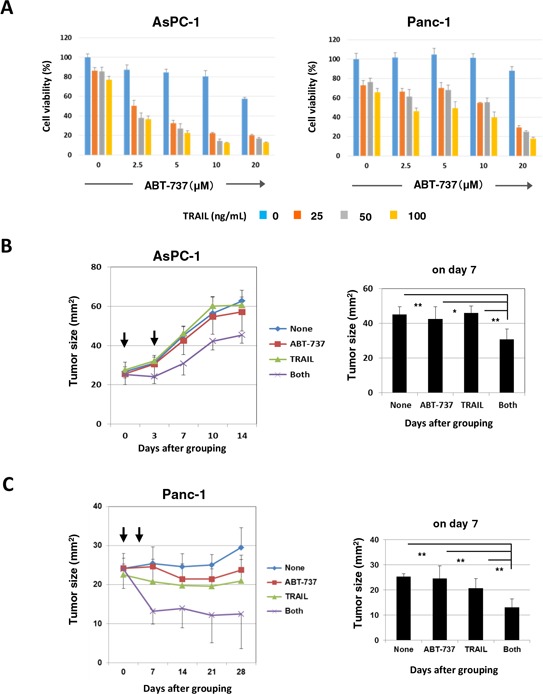
*In vivo* antitumor effect of DTX and ABT-737 on the growth of PC3 cells **A.** AsPC-1 and Panc-1 cells were cultured with the indicated dose of ABT-737. After 48 h, cell viability (%) was assessed using the WST-8 assay. The results are shown as the means ± SD of three wells. **B.** BALB *nu/nu* female mice were inoculated in their right flank with 3 × 10^6^ AsPC-1 cells in Matrigel. When the tumor diameter reached around 5 mm, the mice were pooled and divided into four groups. On days 0 and 3 after grouping, these pancreatic cancer-bearing mice were treated with i.t. injection of TRAIL (1 μg) and/or i.p. injection of ABT-737 (75 mg/kg). As a vehicle control for TRAIL, 50 μL PBS was injected. As a vehicle control for ABT-737, 100 μL DMSO was administered. The tumor size was measured once or twice weekly. Each group contained six mice. **C.** BALB *nu/nu* female mice were inoculated in the right flank with 3 × 10^6^ Panc-1 cells in Matrigel. On day 10, the mice were pooled and divided into four groups and treated similarly. *P < 0.05, **P < 0.01 (ANOVA with Bartlett's test).

We initially compared the *in vitro* antitumor effect of two orally bioavailable Bcl-2 inhibitors, ABT-263 and ABT-199, and found that ABT-263 was able to sensitize human pancreatic cancer cells to TRAIL. However, in xenograft mouse models, we used ABT-737 in place of ABT-263 because oral administration of ABT-263 did not work in another xenograft mice model using human prostate cancer cells and an anti-cancer drug, docetaxel [35], and because ABT-737 can be administered systemically [16]. The combination of TRAIL and ABT-737 synergistically reduced the viability of AsPC-1 and Panc-1 cells *in vitro*, as observed in the case with ABT-263 (Figure 7A). We performed experiments in which tumor-bearing mice were injected intratumorally (i.t.) with TRAIL (1 μg in 50 μL) and/or intraperitoneally (i.p.) with ABT-737 (75 mg/kg) on days 0 and 3 after grouping (Figure 7B and 7C). In both experiments, the combination of TRAIL and ABT-737 significantly suppressed tumor growth on day 7 after grouping compared with the groups treated with either drug separately. Body weight was measured to evaluate the general condition of these mice, but no difference was observed among the four groups (Supplementary Figure S4). These results indicate that Bcl-2 family inhibitors, such as ABT-737, can sensitize TRAIL-resistant human pancreatic cancer cells to TRAIL *in vivo*.

## DISCUSSION

The anti-apoptotic Bcl-2 family of proteins, including Bcl-2 and Bcl-xL, protect cancer cells from apoptosis [10–15]. Several reports have shown that Bcl-2 family proteins contribute to TRAIL resistance [36–39]. Additionally, a report has suggested that knockdown of Bcl-xL or Mcl-1 can induce apoptosis in pancreatic cancer cells [40]. Furthermore, ABT-263 and ABT-737 induce apoptosis in prostate cancer, hepatocellular carcinoma, colon cancer, and glioblastoma when combined with TRAIL [29, 39, 41, 42]. In terms of human pancreatic cancer, ABT-737 and obatoclax, which inhibits Bcl-2, Bcl-xL, and Mcl-1, were shown to enhance the TRAIL sensitivity of pancreatic cancer cell lines [43, 44]. However, these studies were performed using only two pancreatic cancer cell lines, and the *in vivo* antitumor effect was not examined.

In this study, TRAIL sensitivity varied among the nine pancreatic cancer cell lines (Figure 1A). Many mechanisms are involved in TRAIL sensitivity [45]. We examined the expression of DRs and DcRs, but TRAIL sensitivity did not correlate with their expression (Figure 1B). Indeed, DR5, but not DR4, seems to be important in TRAIL sensitivity. We also found that the TRAIL sensitivity was inversely correlated with protein expression of Bcl-xL (Figure 2A). Additionally, knockdown of Bcl-xL by siRNA significantly increased the TRAIL sensitivity of AsPC-1 and Panc-1 cell lines (Figure 5), which is consistent with a previous report that demonstrated that Bcl-xL protects pancreatic adenocarcinoma cell lines from apoptosis via CD95/DR signaling [38]. In this study, we utilized three Bcl-2 family inhibitors: ABT-263 and ABT-737 are inhibitors of Bcl-2, Bcl-xL, and Bcl-w [16–18], whereas ABT-199 inhibits Bcl-2 and Bcl-w, but not Bcl-xL [19]. A comparison of their effects enabled us to evaluate the role of Bcl-xL in TRAIL resistance in cancer cells. The results showed the synergistic or additive antitumor effects of TRAIL and ABT-263 in four TRAIL-resistant cancer cell lines (Figure 2B and 2C). We previously examined the sensitivity of normal epithelial prostate cells to ABT-263 and found a similar sensitivity to ABT-263 in human prostate cancer cell lines [35], suggesting that its effect is not cancer-specific. Alternatively, ABT-737 has been reported to potentiate the TRAIL-induced apoptosis of human pancreatic cancer cells by conformationally changing Bax and disrupting the binding of Bak with Bcl-xL [43]. Furthermore, a survey of gene expression and response to chemotherapy agents in the NCI-60 panel identified Bcl-xL as a major cause of chemoresistance in epithelial cancer cells [46]. This indicates that the protective role of Bcl-xL is not limited to TRAIL-induced apoptosis but also occurs with chemoresistance. Additionally, we recently found that Bcl-xL is responsible for the docetaxel resistance of human prostate cancer cells [35]. Alternatively, without TRAIL, the simultaneous knockdown of Bcl-xL and Mcl-1 induced apoptosis via Bax activation in pancreatic cancer cells [40]. If this were the case, inhibition of both Bcl-xL and Mcl-1 could sensitize TRAIL-resistant pancreatic cancer cells more efficiently than Bcl-xL alone.

In addition to the effect on Bcl-xL, we investigated other mechanisms by which ABT-263 induced or restored the TRAIL sensitivity of pancreatic cancer cells. Several possibilities could be proposed as TRAIL-resistant mechanisms: expression of DcRs, dysfunction of DR4/DR5, loss or down-regulation of FADD or caspase-8, overexpression of c-FLIP, overexpression of anti-apoptotic proteins (e.g., Bcl-2 and Bcl-xL) or mutation of pro-apoptotic proteins (e.g., Bax and Bak), and overexpression of inhibitors of apoptosis (IAP) [36, 37]. Among these possibilities, we examined the effects of ABT-263 on the expression of Bcl-2, Bcl-xL, c-FLIPs, and DR4/DR5 by focusing on two cell lines, AsPC-1 and Panc-1. We found that ABT-263 failed to influence the expression of Bcl-2, Bcl-xL, and c-FLIPs (Figure 6A), but it did reduce the expression of DR5 on only Panc-1 cells (Figure 6B). In this regard, ABT-737 has been reported to increase the expression of DR5 on prostate, renal, and lung cancer cell lines at a transcriptional level as a result of stimulation of NF-κB activity [29]. We tested this possibility and found that PDTC inhibited the increased expression of DR5 on ABT-263-treated Panc-1 cells (Figure 6C). Alternatively, several chemotherapeutic drugs and stress inducers have been reported to increase the expression of DR4 and DR5 on cancer cells [47, 48]. ER stress is induced when protein-folding stress at the ER is generated [49]. ER stress increases the protein expression of DR5 via the unfolded protein response mediator CHOP [31, 32, 34]. Additionally, Bcl-2 inhibitors, such as ABT-737 and obatoclax, induce ER stress in human melanoma cells [50]. Therefore, we determined whether ER stress was involved in the increased expression of DR5 on ABT-263-treated Panc-1 cells, but the result was negative (Figure 6D and 6E). Totally, our results indicate that the NK-κB pathway, but not ER stress, may be involved in increased expression of DR5 on ABT-263-treated Panc-1 cells.

In conclusion, we found that Bcl-xL is responsible for TRAIL resistance in human pancreatic cancer cells and that the Bcl-2 family inhibitors, including ABT-263 and ABT-737, induce/restore the TRAIL sensitivity of pancreatic cancer cell lines both *in vitro* and *in vivo*. The Bcl-2 family of inhibitors could be promising reagents to sensitize human pancreatic cancer cells in DR-targeting therapy.

## MATERIALS AND METHODS

### Cell lines

Seven human pancreatic cancer cell lines (BxPC-3, SW1990, CAPAN-2, CFPAC-1, Panc10.05, AsPC-1, and HPAF-II) were purchased from the American Type Culture Collection (Manassas, VA, USA). Two other human pancreatic cancer cell lines (MiaPaCa-2 and Panc-1) were kindly provided by Dr. K. Takenaga (Shimane University Faculty of Medicine) [51]. These cell lines were maintained in DMEM (Sigma-Aldrich, St. Louis, MO, USA) supplemented with 10% fetal calf serum (Invitrogen, Grand Island, NY, USA) and 20 μg/ml gentamicin (Sigma-Aldrich).

### Cell viability assay

Cell viability was analyzed using the WST-8 assay (Nacalai Tesque, Kyoto, Japan). At the end of the incubation period, 10 μl WST-8 solution was added to each well, and the plates were incubated for an additional 3 h. Absorbance in each well was measured at 560 nm using a microplate reader (Beckman Coulter, Brea, CA, USA).

### Detection of DR and DcR expression on cells

To examine the expression of DR4 (CD261) and DR5 (CD262), cells were incubated with either anti-DR4 (eBioscience, San Diego, CA, USA) or anti-DR5 (eBioscience), followed by staining with FITC-conjugated goat anti-mouse IgG (H+L) (KPL, Gaithersburg, MD, USA). To examine the expression of DcR1 (CD263) and DcR2 (CD264), cells were stained with either FITC-conjugated anti-DcR1 (CD263) (GeneTex, Irvine, CA, USA), or FITC-conjugated anti-DcR2 (CD264) (GeneTex). For these incubations, isotype-matched FITC-conjugated mouse IgG1 was used as a control. Analysis was performed using a FACSCalibur flow cytometer (Becton Dickinson, Franklin Lakes, NJ, USA).

### Immunoblotting

Cells were lysed with a mammalian protein extraction reagent (M-PER; Thermo Scientific, Rockford, IL, USA) containing a protease-inhibitor cocktail (Nacalai Tesque). Equal amounts of protein were resolved on 4–12% gradient or 12% SDS-PAGE gels and transferred to polyvinylidene fluoride membranes. The membranes were blocked and the blots incubated with the following primary antibodies: anti-Bcl-2 (sc-492; Santa cruz biotechnology (SCB), Dallas, Texas, USA, anti-Bcl-X_S/L_ (#633901; BioLegend, San Diego, CA, USA), anti-Mcl-1 (sc-819; SCB), anti-FLIP_S/L_ (sc-5276; SCB), anti-DR5 (#8074; Cell signaling technology (CST), Danvers, MA, USA), anti-CHOP (#2895; CST), anti-caspase-3 (#9668; CST), anti-caspase-8 (M032–3; Medical and Biological Laboratories, Nagoya, Japan), anti-caspase-9 (#9508; CST), anti-Bid (#2002; CST), anti-β-actin (BioLegend), or anti-α-tubulin (SCB). After washing, room temperature incubation of membranes for 30 min with either goat anti-rabbit or goat anti-mouse alkaline phosphatase-conjugated secondary antibodies (Invitrogen) was used to detect the primary antibodies. Protein bands were visualized using CDP-star chemiluminescence and imaged using an ImageQuant LAS-4000 system (FujiFilm, Tokyo, Japan).

### Apoptosis assay

Apoptosis was assessed using the Annexin V-FITC Apoptosis Detection Kit (BioVision, Mountain View, CA, USA) and PI. Each caspase inhibitor (20 μM), or the same volume of DMSO as a vehicle control, was added 1 h before the addition of TRAIL. After staining with annexin V-FITC/PI, flow cytometric analysis was performed. Analysis was performed using a FACSCalibur flow cytometer.

### Reagents

For inhibition assays, the following inhibitors were added 1 h before the addition of TRAIL: pan-caspase inhibitor Z-VAD-FMK (Enzo Life Sciences, Farmingdale, NY, USA), caspase-8 inhibitor Z-IETD-FMK (R&D Systems, Minneapolis, MN, USA), and caspase-9 inhibitor Z-LEHD-FMK (R&D Systems). Thapsigargin was purchased from Nacalai Tesque. PDTC was purchased from Calbiochem (La Jolla, CA, USA).

### Confocal imaging

Cancer cells were seeded onto round microscope cover glasses in 24-well plates and cultured with TRAIL (25 ng/mL) and/or ABT-263 (1 μM) for 12 h. After incubation with Hoechst 33342 (5 μg/ml) and MitoTracker Red (20 nM) (#9082; CST) for 30 min, cells were fixed and permeabilized with 3% formalin and 1% Triton X, respectively, and stained with anti-Bax antibody (D2E11) (#5023; CST) followed by Alexa Fluor 488-conjugated anti-rabbit IgG F(ab’)2 fragment (CST). The cover glasses were then placed on slides with 4 μl of mounting medium for fluorescence (Vectashield; Vector Laboratories, Inc., Burlingame, CA, USA). Confocal imaging was performed using an Olympus FV1000-D laser scanning microscope (Olympus, Tokyo, Japan).

### Transfection of small interfering RNA (siRNA)

Transfection of siRNA was performed using Lipofectamine^TM^ RNAiMAX (Invitrogen, Grand Island, NY, USA) according to the manufacturer's instructions. Bcl-2 and Bcl-xL siRNAs were purchased from Santa Cruz Biotechnology (Santa Cruz, CA, USA) and Invitrogen, respectively. Control siRNA (#6568) was purchased from Cell Signaling Technology (Danvers, MA, USA). The transfected cells were used for the experiments 3 days after siRNA transfection.

### *In vivo* xenograft models

Female BALB *nu/nu* mice, purchased from CLEA Japan (Tokyo, Japan), were maintained under specific pathogen-free conditions. The protocol was approved by the Committee on the Ethics of Animal Experiments of the Shimane University Faculty of Medicine (Permit Number: IZ26–103). All efforts were made to minimize suffering. Mice were inoculated in the right flank with 3 × 10^6^ pancreatic cancer cells and Matrigel (Japan BD Biosciences, Tokyo, Japan) at a 1:1 volume ratio in a total volume of 100 μL. When the tumor diameter reached approximately 5 mm, the mice were pooled and divided into four groups. On days 0 and 3 after grouping, the pancreatic cancer-bearing mice were administered an intratumoral (i.t.) injection of TRAIL (1 μg) and/or an intraperitoneal (i.p.) injection of ABT-737 (75 mg/kg). As a vehicle control for TRAIL, 50 μL PBS was injected. As a vehicle control for ABT-737, 100 μL DMSO was administered. The tumor size was measured once or twice weekly. Each group contained six mice.

### Statistical analysis

Data were evaluated statistically using an unpaired two-tailed Student's *t*-test or an ANOVA together with Bartlett's test. A *P*-value < 0.05 was considered to indicate significance.

## SUPPLEMENTARY FIGURES


