# Supplementary material for: Bcl-xL inhibition by molecular-targeting drugs sensitizes human pancreatic cancer cells to TRAIL
Source: Oncotarget. 2015 Oct 19;6(39):41902–15. doi: 10.18632/oncotarget.5881 (PMC4747197; doi:10.18632/oncotarget.5881)
Supplement: Supplementary file 1 [file oncotarget-06-41902-s001.pdf]

SUPPLEMENTARY FIGURES

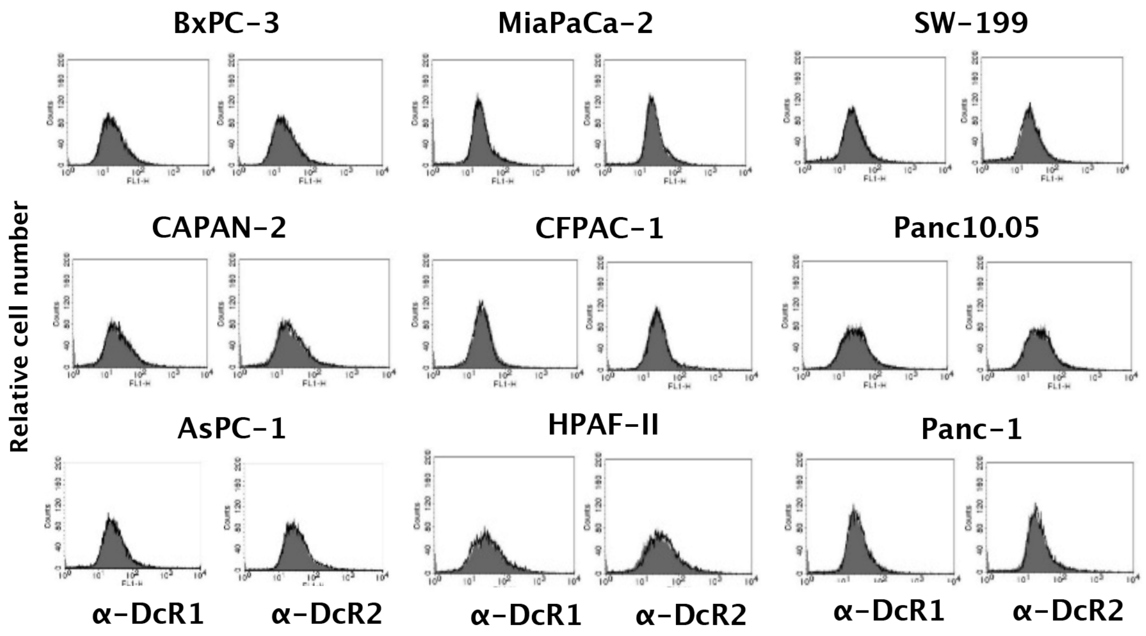

Supplementary Figure S1: Expression of DcRs on nine pancreatic cancer cell lines. The expression of DcRs on nine cell lines was examined by flow cytometry. The line represents staining with FITC-conjugated mAb specific to either DcR1 or DcR2. Solid gray represents staining with FITC-conjugated anti-mouse IgG alone. Solid gray represents staining with isotype-matched FITC-conjugated anti-mouse IgG.

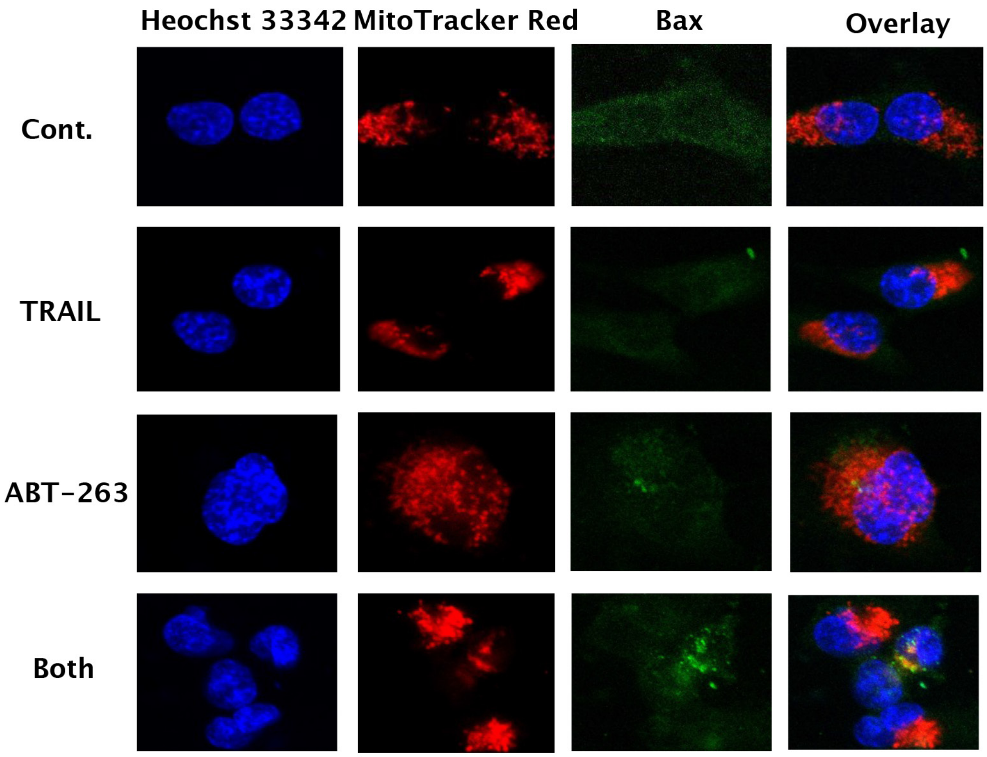

Supplementary Figure S2: Translocation of Bax to the mitochondria in Panc-1 cells treated with both TRAIL and ABT-263. Panc-1 cells were with TRAIL (50 ng/mL) and/or ABT-263 (5 µM) for 12 h. Cells were examined by confocal imaging as in Figure 4D. Imaging revealed nuclei (blue), mitochondria (red), and Bax (green). Yellow represents Bax that localized to the mitochondria.

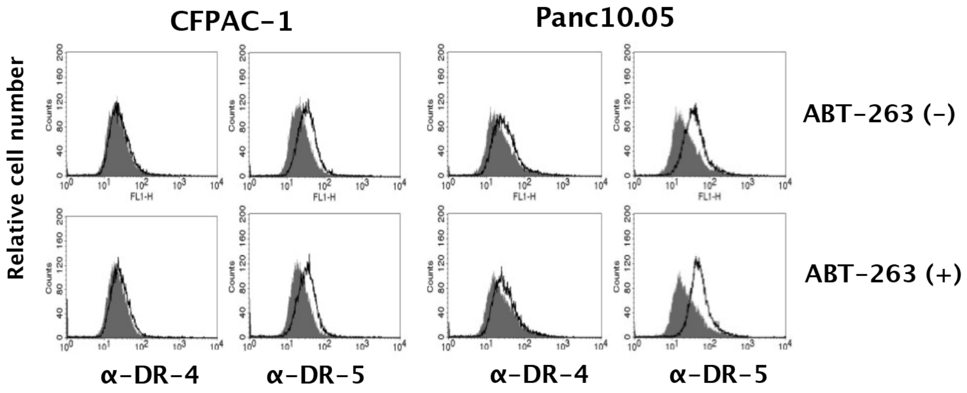

Supplementary Figure S3: Expression of DRs on CFPAC-1 and Panc10.05 cell lines treated with ABT-263. After treatment with ABT-263 (2.5 µM) for 24 h, the expression of DRs on CFPAC-1 and Panc10.05 cells was examined by flow cytometry. The line represents staining with mAb specific to either DR4 or DR5, followed by a FITC-conjugated secondary antibody. Solid gray represents staining with FITC-conjugated anti-mouse IgG alone.

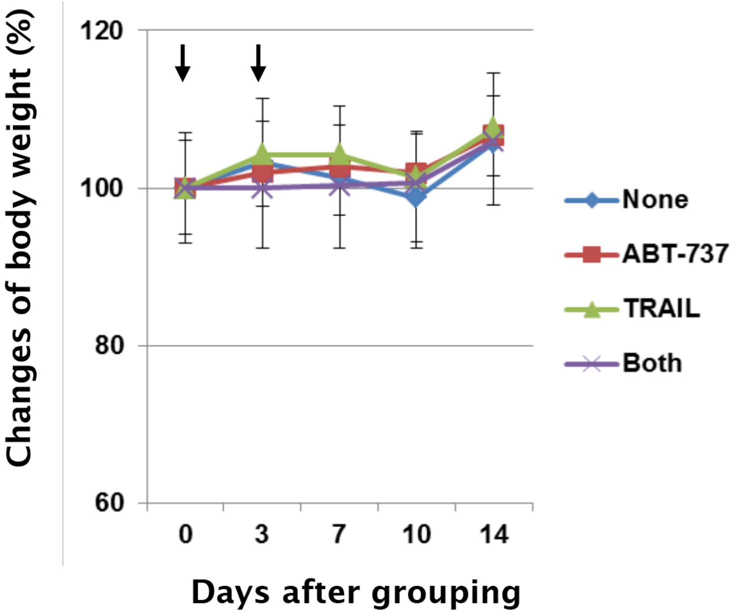

Supplementary Figure S4: Kinetic changes of body weight of AsPC-1-bearing mice treated with TRAIL and/or ABT-737. BALB *nu/nu* female mice were inoculated in the right flank with 3×10^6 AsPC-1 cells and divided into four groups. On days 0 and 3 after grouping, pancreatic cancer-bearing mice were treated with i.t. injection of TRAIL (1 μg) and/or i.p. injection of ABT-737 (75 mg/kg). Body weight was measured twice weekly. Each group contained six mice.
